# Assessing direct healthcare costs when restricted to self-reported data: a scoping review

**DOI:** 10.1186/s13561-021-00330-2

**Published:** 2021-09-16

**Authors:** Samira B. Jabakhanji, Jan Sorensen, Gintare Valentelyte, Lee Ann Burke, Brendan McElroy, Aileen Murphy

**Affiliations:** 1 Healthcare Outcomes Research Centre, Royal College of Surgeons in Ireland, Dublin, County Dublin Ireland; 2 Department of Economics, Cork University Business School, University College Cork, Cork, County Cork Ireland

**Keywords:** Costs, Resources, Surveys, Scoping review, Ireland

## Abstract

**Background:**

In the absence of electronic health records, analysis of direct healthcare costs often relies on resource utilisation data collected from patient-reported surveys. This scoping review explored the availability, use and methodological details of self-reported healthcare service utilisation and cost data to assess healthcare costs in Ireland.

**Methods:**

Population health surveys were identified from Irish data repositories and details were collated in an inventory to inform the literature search. Irish cost studies published in peer-reviewed and grey sources from 2009 to 2019 were included if they used self-reported data on healthcare utilisation or cost. Two independent researchers extracted studies’ details and the PRISMA-ScR guidelines were used for reporting.

**Results:**

In total, 27 surveys were identified containing varying details of healthcare utilisation/cost, health status, demographic characteristics and health-related risk and behaviour. Of those surveys, 21 were general population surveys and six were study-specific ad-hoc surveys. Furthermore, 14 cost studies were identified which used retrospective self-reported data on healthcare utilisation or cost from ten of the identified surveys. Nine of these cost studies used ad-hoc surveys and five used data from pre-existing population surveys. Compared to population surveys, ad-hoc surveys contained more detailed information on resource use, albeit with smaller sample sizes. Recall periods ranged from 1 week for frequently used services to 1 year for rarer service use, or longer for once-off costs. A range of perspectives (societal, healthcare and public sector) and costing approaches (bottom-up costing and a mix of top-down and bottom-up) were used. The majority of studies (*n* = 11) determined unit prices using multiple sources, including national healthcare tariffs, literature and expert views. Moreover, most studies (*n* = 13) reported limitations concerning data availability, risk of bias and generalisability. Various sampling, data collection and analysis strategies were employed to minimise these.

**Conclusion:**

Population surveys can aid cost assessments in jurisdictions that lack electronic health records, unique patient identifiers and data interoperability. To increase utilisation, researchers wanting to conduct cost analyses need to be aware of and have access to existing data sources. Future population surveys should be designed to address reported limitations and capture comprehensive health-related, demographic and resource use data.

**Supplementary Information:**

The online version contains supplementary material available at 10.1186/s13561-021-00330-2.

## Introduction

In the absence of electronic health records (EHR) linkable through unique patient identifiers, researchers must rely on collecting or using secondary healthcare-user data to assess healthcare utilisation and cost. This is the case in Ireland, which is one of seven Organisations for Economic Co-operation and Development countries that lack a comprehensive system of unique patient and provider identifiers [1]. Despite efforts to roll out Individual Health Identifiers and implement EHRs, data are not regularly captured in national statistics monitoring health system performance, patient safety or public health. Overall, employed systems lack interoperability, change is slow and often data are fragmented and records inaccessible [1–3], meaning that records in Ireland cannot be linked, yielding barriers to fully developing and using top-down costing approaches. Accordingly, it is difficult to obtain comprehensive descriptions of cost and resource use at a local or national level to inform policy and practice planning to prioritise prevention and match patient needs. While these barriers are not unique to Ireland they rarely occur cumulatively [1]. Consequential resource allocation decisions can lead to suboptimal patient care [4].

Cost analyses of healthcare resources provide information about current allocation and inform debates about rational and efficient future redistribution of resources for prevention and treatment of ill-health, complementing clinical evidence in cost-effectiveness analyses for example where used [5]. Reliable and detailed data could facilitate tailored resource allocations to individual patient and population groups, based on which the staffing and funding of healthcare services can be organised to meet current and future healthcare demands. This requires the identification of health threats, healthcare need and cost drivers of healthcare in the first place, all of which can then be addressed through intervention studies (trials) to identify cost-effective strategies for health policy and clinical practice.

For planning and evaluation, it is common practice to use the principle of attributable costs to establish the costs related to risk factors or long-term cost of a disease. The attributable cost approach requires that the cost of healthcare provided to two groups of individuals can be established by aggregating different sources of data about resource use and cost for each individual [6]. To attribute costs, data are needed in relation to the individual health status (e.g. disease history and outcomes; anthropometric measures; laboratory data; quality of life) and healthcare utilisation (e.g. type, frequency and time of health service use). For a comprehensive identification of cost drivers and needs, additional data requirements include demographic information (sex; age), information on health-related risk and behaviour (genetic predisposition; socio-economic status; environmental factors; lifestyle) and, to integrate a societal perspective, data on labour force participation (absenteeism; presenteeism) and opportunities (educational outcomes) are needed. To maximise accuracy, these various data from an individual should be linked, and available over time. Ideal sources include patient record files and (disease-specific) national registries containing additional details on health-related risk factors, behaviours, and labour force participation [7].

While method guides exist for some jurisdictions, primarily for conducting health economic evaluations, inconsistencies persist with different costing methods and approaches yielding different results. In response, there are calls for more consistent and efficient costing methods [5]. Generally, health system costs can be assessed using top-down approaches (gross costing) which rely on centralised data repositories, such as hospital patient record files collected prospectively. Alternatively bottom-up approaches (micro or activity-based costing) using data collected directly from healthcare users, often through surveys, censuses or diaries. The advantages of top-down costing include the requirement of fewer resources and provision of better opportunities for generalisation, but at the expense of precision when informing economic evaluations of interventions. Bottom-up costing is more laborious, but provides greater insights into the relationship between activity and cost characteristics, the economies of scale, and the relative costs of different activities and variation in patient-related cost [8, 9].

Thus, when analysing data on healthcare utilisation in jurisdictions without HER or linked systems, like Ireland, researchers have limited options. Individual data may be collected from ‘top-down’ perspective from individual service providers, incurring a high administrative and resource burden. Alternatively, self-reported user data may be used. Often this includes cross-sectional and longitudinal quantitative data collected from general population health surveys or disease-specific surveys and extrapolated to population level, from a ‘bottom-up’ approach. In comparison, trial data from intervention studies typically involve small disease- and location-specific populations and are less representative of and generalisable to regional or national populations. While data on healthcare utilisation and cost measures gathered from quantitative surveys potentially lack precision, they frequently represent the best available data to assess healthcare costs in jurisdictions like Ireland.

While the existence of this issue is commonly acknowledged, here we explore the extent to which general population health surveys are used to overcome it when conducting cost analyses. In doing so we firstly identify the self-reported health service utilisation and cost surveys/datasets available, and consider their suitability for costing direct healthcare use. Secondly, we conduct a scoping review to identify and examine Irish costing studies (including cost analysis, cost estimation and cost of illness) that applied these surveys/datasets to calculate direct healthcare costs.

## Methods

An inventory of Irish population health surveys was developed and a scoping review [10, 11] was conducted to map the methodologies and data sources used in Irish studies of direct healthcare costs.

### Search strategy

Initially, the Irish Social Science Data Archive, safefood, Central Statistics Office and the Department of Health websites and repositories were searched to identify population surveys that contain data on health, healthcare utilisation or healthcare-related cost. To obtain additional information for each of the identified surveys, survey-specific websites and original questionnaires were reviewed. The list of surveys informed the subsequent literature search.

A PubMed literature search, conducted from 16th to 18th October 2019, identified studies published between October 2009 and October 2019 that assess self-reported healthcare use and cost data in the Republic of Ireland. Initially, the MeSH Term ‘cost’ was combined with ‘questionnaire’ [MeSH]. Subsequently, ‘cost’ [MeSH] was combined with the full name and abbreviations of the identified population health surveys (Supplementary Material 1). We also searched grey literature for reports and scientific papers not listed on PubMed. Specifically, the Irish Social Science Data Archive, safefood, Central Statistics Office and the Department of Health websites and repositories were searched for healthcare-related publications.

### Analysis

For each population health survey, information was extracted on data collection tools, sample characteristics and the type of healthcare services for which data were collected. Additionally, availability of demographic information, socio-economic information, health status and lifestyle variables from surveys were recorded. This information is collated in an inventory.

For the scoping review of published studies, two researchers independently reviewed the abstracts of all retrieved citations. Initial charting included basic information of the underlying dataset and availability of data on healthcare utilisation or costs. Full-text studies and reports were retained if they (1) used survey or otherwise self-reported data on healthcare utilisation or cost, including patients’ direct out-of-pocket costs, and (2) used these data to assess direct healthcare costs. Whereby direct healthcare costs only refer to resources used in the healthcare sector (excluding resources used in other sectors, such as social care) [12]. The following exclusion criteria were applied: Publication outside the study period and region; studies reporting indirect healthcare costs only; studies using simulations, decision-trees or Markov-models only; studies applying primary data from other jurisdictions to the Irish setting; and study protocols. Intervention studies reporting treatment costs (clinical trials) were excluded as these are often not representative of the Irish population and data not always self-reported. Patient registry studies were excluded as these data are often not self-reported.

Studies qualifying for inclusion were reviewed in full and information of data sources and costing methodologies extracted. In particular, details of perspectives and costing methods were identified, areas of health services for which costing was undertaken and reflections on costing methods and datasets were synthesised. Methodological quality and risk of bias were not assessed as this not a common component of scoping reviews [10]. Findings were reported using the PRISMA-ScR guidelines [13, 14].

## Results

### Survey inventory

We identified 21 population surveys or survey tools that contain data on health status, healthcare utilisation or healthcare cost, available from Irish data repositories (Table 1). The literature review identified six additional surveys that researchers used on an ad-hoc basis to inform their assessment of direct healthcare costs (included on Table 2).

**Table 1:** Inventory of Irish surveys containing questions on healthcare utilisation or health status

Dataset	Year(s) of data collection	Sample size	Population	Healthcare utilisation data							Additional information				Source
				Primary care	Specia-list services	Medi-cinal pro-ducts	Hospital out-patient services	Emergency visits	Hospital inpatient admission	Hospital length of stay	Demo-graphic	Socio-eco-nomic	Health status	Life-style	
AITHS	2007	8430	National, Traveler population	X	X			X		X	X	X	X	X	ISSDA
Census of Population	1971, 1979, 1981, 1986, 1991, 1996, 2002, 2006, 2011, 2016	4.76 Mil	Population								X	X	X		CSO/ ISSDA
CoHeart	2000–2001, 2005–2006	1084	Regional - cardiac population in West of Ireland	X		X	X				X	X	X	X	ISSDA
CSPPA	2009	4122	National, children & youth								X	X		X	ISSDA
European Social Survey	2002, 2004, 2006, 2008, 2010, 2012, 2014, 2016, 2018		National								X	X	X		Europeansocialsurvey.org / ISSDA
EU-SILC	2003–2017	11,130	National	X		X			X		X	X	X	X	Eurostat/ CSO
Growing Up in Ireland	GUI 08: 2008–2009, 2011, 2013, 2016, 2017–2018	11,134	National, children born in 2008	X	X	X	X	X		X	X	X	X	X	ISSDA
	GUI 98: 2008, 2011, 2013, 2017	8570	National, children born in 1998	X	X	X	X	X		X	X	X	X	X	ISSDA/ safefood
Healthy Ireland	2014–2015, 2015–2016, 2016–2017, 2018–2019	6529	National		X		X	X	X	X	X	X	X	X	ISSDA/ DOH
Household Budget Survey	1987, 1999–2000, 2004–2005, 2009–2010, 2015–2016	6839	National	X		X				X	X	X			ISSDA
Insight ‘07	2007	3517	National	X	X		X	X			X	X	X	X	ISSDA
Irish Health Survey, QNHS	2001, 2002, 2004, 2007, 2010, 2013–2016	10,323	National	X	X	X			X	X	X	X	X	X	CSO
INTUS	2005	1203	National								X	X	X	X	ISSDA
ISSHR	2006	7668	National	X	X	X	X				X	X	X		ISSDA
Irish EQ-5D-5L	2015–2016	1160	National								X	X	X		ISSDA
NANS	2008–2010	1500	National								X			X	UCC / UCD
NPWDS	2006	2711	National	X	X	X					X	X	X		ISSDA
School Leavers Survey	1980–2007	2025	National								X	X	X		ISSDA
SPHERE^a^	2003–2008	960	Regional (Belfast, Dublin & Galway), coronary heart disease	X		X	X	X	X		X	X	X	X	ISSDA
SLÁN	1998; 2002, 2007	10,364	National	X			X		X	X	X	X	X	X	ISSDA
Sports & PE, QNHS	2013	15,860	National								X	X		X	ISSDA
TILDA	2009–2016	6279	National, adults aged + 50	X	X	X	X	X	X	X	X	X	X	X	ISSDA/ TCD

This table presents an inventory of Irish datasets and surveys identified from the websites and repositories of the Irish Social Science Data Archive (ISSDA), safefood, Central Statistics Office (CSO) and the Department of Health (DOH)

*AITHS* All Ireland Traveller Health Study, *CSPPA* Children’s Sport Participation and Physical Activity Study, *EU-SILC* European Union Survey of Income and Living Conditions; Insight ‘07, National Survey of Consumer Satisfaction, *INTUS* Irish National Time-Use Survey, *ISSHR* Irish Study of Sexual Health and Relationships, *Irish EQ-5D-5L* Irish EQ-5D-5L Survey, *NANS* National Adult Nutrition Survey, *NPWDS* National Psychological Wellbeing and Distress Survey, *QNHS* Quarterly National Household Survey – quarterly survey that contains modular additions, *SPHERE* Secondary Prevention of Heart Disease in General Practice, *SLÁN* Survey on Lifestyle and Attitude to Nutrition, *Sports & PE* Sports & Physical Exercise module in QNHS, *TCD* Trinity College Dublin, *TILDA* The Irish Longitudinal study on Ageing, *UCC* University College Cork, *UCD* University College Dublin, X indicates presence variables

^a^Included healthcare cost data (measured in monetary amounts)

**Table 2:** Summary of methodologies used to assess direct healthcare costs using self-reported data

Study	Disease / condition	Tool/dataset to collect self-reported data on healthcare resources	Standardised tools for sub-group analysis of costs by health status	Healthcare resources included in cost estimates: Self-reported	Healthcare services included in cost estimates: Other data sources^b^	Recall periods of self-reported healthcare resources	Where do unit prices come from	Cost estimation: 1. Extrapolation to national level 2. Perspective 3. Costing method 4. Additional details
Carney et al. (2018) [15]	Multiple sclerosis; relapse of multiple sclerosis	Ad-hoc dedicated survey (n = 594)^d^: CSRI administered to patients registered with national patient association	EQ 5D-5L; EQ-VAS	Inpatient hospital care, nursing home, rehabilitation, respite care, radiological investigations, laboratory investigations, other investigations, outpatient services, emergency department visits, primary care, specialist services, physiotherapy, occupational therapy, speech therapy, aids	Medication (literature)	Varying: 1 week, 6 months or 12 months; full duration of disease; during last period of relapse	National casemix/DRGs, national salary scales, national literature, Irish HC professionals (expert opinion), Irish central statistics office, other company and service price lists	1.yes 2.Societal 3. Bottom-up 4. Cost of appliances annualised with 4% discount rate
Ceilleachair et al. (2017) [16]	Colorectal cancer	Ad-hoc dedicated survey (*n* = 497): patient economic impact questionnaire ^e^	Stratification by disease stage (tool not mentioned)	Out of pocket costs for primary care, medication, appliances	None	Since diagnosis	Survey specifically asking about costs	1. No 2. Patient 3. Bottom-up
Connolly et al. (2014) [17]	Dementia	Use of existing data from ECAD study in *Gillespie* et al. (2013)	Dependence Scale; Disability Assessment for Dementia scale; Mini-Mental State Examination scale; Neuropsychiatric Inventory	Primary care, community care, medication	GP services, respite care, nurse, physiotherapist, psychologist, chiropodist, occupational therapist, outpatient consultations, emergency admissions, medication (prior publications: Enhancing Care in Alzheimer’s disease (ECAD) study (Gallagher et al., 2010; Gillespie et al., 2012).	Varying: 6 months, 12 months	National casemix/DRGs, national salary scales, national literature, Irish central statistics office	1. Yes 2. Societal 3. Top-down and bottom-up
Dee et al. (2015) [18]	Overweight and obesity (body mass index > 25)	SLÁN 2007 & QNHS Q3	/	GP services, outpatient costs *(taken from Doherty* et al.*, 2013* [19]	GP services (Doherty et al. 2012 using SLAN), hospital inpatient care & day case care (HIPE in ROI, HIS in NI), medication (PCRS in ROI and BSO in NI)	12 months	National casemix/DRGs, national pharma database, national salary scales, social welfare data, national literature, Irish central statistics office	1. Yes 2. Societal 3. Bottom-up
Doherty et al. (2013) [19]	Overweight and obesity (body mass index > 25)	SLÁN 2007 & QNHS Q3	General Health scale (5-fold)	GP services, inpatient hospital care, hospital day case services	None	12 months	Average consumer fees for GP care; National literature	1. Yes 2. Healthcare services^a^ 3. Bottom-up
Doherty and O’Neill (2014) [20]	Osteoarthritis, rheumatoid arthritis amongst older adults	TILDA	No tool mentioned	GP services, outpatient hospital services, emergency dept. visits, hospital inpatient care	None	12 months	National literature	1. Yes 2. Healthcare services^a^ 3. Bottom-up
Fogarty et al. (2014) [21]	Multiple sclerosis	Ad-hoc Dedicated Survey (*n* = 214): CSRI administer, patients in St. Vincent’s University Hospital, Dublin	EQ-5D-5L; Extended Disability Status Scale	Inpatient admissions, rehabilitation, nursing home care, outpatient care, primary healthcare visits, respite care, laboratory investigations, radiological investigations, other investigations, medication, mobility and other living aids	None	Varying: 1 week, 1 month, 6 months, 12 months; full duration of disease	National casemix/DRGs, national pharma database, national salary scales, social welfare data (national home scheme support), national literature, Irish HC professionals (expert opinion and laboratory and finance departments), Irish central statistics office, other company and service price lists, patient estimates	1. Yes 2. Societal 3. Bottom-up
Gannon et al. (2013) [22]	Chronic pain (non-cancer)	Ad-hoc Dedicated PRIME Survey (*n* = 100): CSRI administer, patients in West of Ireland	No tool mentioned	Inpatient hospital care, outpatient visits to specialty care, outpatient visits to primary care, emergency room visits, ambulance costs, family medical practice nurse, psychologist, psychiatrist, public health nurse, occupational therapy, physiotherapy, chiropractor, acupuncture, homeopathy, prescription medication, mobility equipment	None	12 months	National casemix/DRGs, national pharma database, national salary scales, Irish HC professionals (expert opinion and laboratory and finance departments), Irish central statistics office	1. Yes 2. Partial societal^a^ 3. Bottom-up 4. Average patient costs across all services and individual patient costs by type of service calculated
Gillespie et al. (2013) [23]	Alzheimer’s disease & mild cognitive impairment in older adults	ECAD study: Ad-hoc dedicated structured caregiver questionnaire (n = 100); including Resource Utilisation in Dementia Lite Instrument	Dependence Scale; Disability Assessment for Dementia scale; Mini-Mental State Examination scale; Neuropsychiatric Inventory	GP services, inpatient hospital visits, outpatient clinic consultations, emergency dept. visits, respite care, registered nurse, physiotherapist, psychologist, chiropodist, occupational therapist	None	6 months	National casemix/DRGs, national salary scales, national literature, Irish central statistics office	1. No 2. Partial societal^a^ 3. Bottom-up
Perry et al. (2017) [24]	Childhood Obesity	GUI cohort ‘98	No tool mentioned	GP services	Hospital inpatient and day care costs, length of stay, mental health outpatient services, medication, specialist services including physiotherapy, psychologists, dieticians, nurses and administrative staff	12 months	National casemix/DRGs, national pharma database, staff costs of specialist childhood obesity unit, national literature, Irish healthcare services (primary care and hospitals), Irish central statistics office (National Employment Survey; National statistics)	1. Yes 2. Public healthcare 3. Top-down and bottom-up
Petrou et al. (2009) [25]	Extremely Preterm Birth (20–25 weeks)	EPICure Study: Postal questionnaire completed by parents (*n* = 331)	15-item Health Utilities Index (HUI2 & HUI3 health status classifications) and various standard tools to assess health and disability status	Hospital inpatient service, hospital day care services, community health services, prescription medication	None	12 months	UK national reference costs; National compendium by the Personal Social Services Research Unit at the University of Kent provided community health and social services unit costs	1. No 2. Public sector 3. Bottom-up 4. UK economic evaluation guidelines used
Petrou et al. (2013) [26]	Extremely Preterm Birth (20–25 weeks)	EPICure Study: Postal questionnaire completed by parents (n = 331) ^c^	15-item Health Utilities Index (HUI2 & HUI3 health status classifications) and various standard tools to assess health and disability status	Hospital inpatient care, hospital day care, outpatient services, community health services, prescription medication	None	12 months	UK national reference costs; National compendium by the Personal Social Services Research Unit at the University of Kent provided community health and social services unit costs	1. No 2. Public sector 3. Bottom-up 4. UK economic evaluation guidelines used
Raftery et al. (2012) [27]	Chronic pain (non-cancer)	Dedicated telephone interview - CSRI; sample (*n* = 140) drawn from PRIME Study;	Chronic pain grade questionnaire to assess age-standardised costs in each pain grade	GP services, emergency dept. visits, inpatient hospital stays, outpatient appointments, prescription & non-prescription medication, complementary and alternative therapies (including psychologists, physiotherapy, chiropractor)	None	12 months	National casemix/DRGs; Irish healthcare services	1. Yes 2. Partial societal^a^ 3. Bottom-up
Richardson et al. (2012) [28]	Polypharmacy in adults over 50	TILDA	General Health scale (5-fold)	Medication, food supplements	None	Daily or weekly use at time of interview	National pharma database	1. Yes 2. Healthcare 3. Bottom-up

*CSRI* Client Service Receipt Inventory, *ECAD*,Enhancing care in Alzheimer’s disease, *EPICure* EPICure study, *GP* General Practitioner, *GUI* Growing Up in Ireland, *QNHS* Quarterly National Househod Survey, *PRIME* Prevalence, Impact and Cost of Chronic Pain, *SLÁN* Survey of Lifestyle, Attitudes and Nutrition in Ireland, *TILDA* The Irish Longitudinal study on Ageing

^a^implied, not explicit

^b^ other data sources mainly were healthcare-provider reported data, such as patient registry data collected in the Hospital In-Patient Enquiry system, disease-specific networks (e.g. Irish Cancer Society) and pharmaceutical registers. A minority of data were taken from previously published literature

^c^Applied UK costs to Irish resource used data

^d^ Authors adapted surveys used in previous empirical studies for the Irish study population

^e^ The patient economic impact questionnaire was a study-specific questionnaire developed by the research team, informed by existing instruments, interviews and focus group discussions with survivors, and consultation with health professionals

References in table

Gallagher, D., Ni Mhaolain, A., Coen, R., Walsh, C., Kilroy, D., Belinski, K., & Lawlor, B. (2010). Detecting prodromal Alzheimer’s disease in mild cognitive impairment: utility of the CAMCOG. International Journal of Geriatric Psychiatry, 25, 1280–1287

Gillespie, P., O’Shea, E., Cullinan, J., Lacey, L., Gallagher, D., Ni Mhaolain, A., & Lawlor, B. (2012). The effects of dependence and function on costs of care for Alzheimer’s disease and mild cognitive impairment in Ireland. International Journal of Geriatric Psychiatry

Doherty E, Dee A, O’Neill C. Estimating the amount of overweight and obesity related health-care use in the republic of Ireland using SLAN data. Econ Soc Rev. 2012;43:227–50

The earliest of these surveys started collecting Irish health data in the 1970s (Census of Population), and approximately half include repeated longitudinal or cross-sectional data collection. Sample sizes vary, ranging from 100 participants in an ad-hoc survey (e.g. in the Enhancing Care in Alzheimer’s disease (ECAD) study) to thousands of participants in the nationally representative longitudinal surveys, (e.g. Growing Up in Ireland (GUI) ‘98 (*n* = 8570) and ‘08 cohorts (*n* = 11,134); the Irish Longitudinal Study on Ageing (TILDA) cohort (*n* = 6279)); cross-sectional repeated surveys (Irish Health Survey (*n* = 10,323), EU-SILC-Ireland (*n* = 11,130), etc.); and national single-use surveys (All Ireland Traveller Health Study (*n* = 8430) and Irish Study of Sexual Health and Relationships (*n* = 7668)).

Of those surveys assessing healthcare service use (*n* = 16), most include data on primary care use (*n* = 14); more than half provide information on specialist services (n = 11), medicinal products (n = 10) or hospital outpatient service use (*n* = 9). Whereas fewer collect data on emergency department visits (n = 7), hospital inpatient admissions (*n* = 6) and number of hospital bed days (n = 7). Almost all surveys provide demographic (*n* = 24) and socio-economic information (n = 24), many reported on the health status of participants (*n* = 19) and lifestyle variables (n = 14).

### Scoping review

The literature search identified 247 abstracts and reports, 165 of which were removed during initial screening and 68 were excluded after full-text review. (For a full list of search terms and results and an overview of the screening process see Supplementary Materials 1 and 2.) After applying the eligibility criteria, 11 peer-reviewed publications and two reports were retained [15–18, 20–28]. Furthermore, one additional study was included from cross-referencing [19].

### Summary of studies

The reviewed full-texts cover the full search period (2009–2018) across a range of eight conditions. Of the 27 surveys identified, 10 are utilised in the following studies.

*Fogarty* et al. (2014) examine direct and indirect 2012-costs (various perspectives) related to Multiple Sclerosis, three disease subtypes and associated disability categories, in 214 patients sampled from a specialist outpatient clinic in Ireland’s capital Dublin. Additionally, *Carney* et al. (2018) measure 2015-societal costs (direct, indirect, intangible and relapse costs) of multiple sclerosis in a nationally representative sample of multiple sclerosis patients (*n* = 594) sampled from a patient registry and through national press and online media.

*Ceilleachair* et al. (2017) investigate various direct and indirect out-of-pocket-costs (2008) in 497 colorectal cancer survivors identified through the National Cancer Registry from their initial diagnosis through treatment and including their initial follow-up approximately 1 year after diagnosis.

*Gillespie* et al. (2013) study the formal and informal cost of care (2008) related to patient dependence and function in patients with Alzheimer’s disease and amnestic mild cognitive impairment, using data from the ECAD study that were collected from the caregivers of 100 community dwelling dementia patients in a memory clinic in Dublin. Furthermore, *Connolly* et al. (2014) assess the costs of dementia in 2010 related to informal care, health and social care, long-stay care and mortality, performing secondary analysis of the ECAD study data and merging this with (unlinked) national hospital and psychiatric inpatient data.

Accounting for the relationship between weight status and various high-cost health conditions, *Doherty* et al. (2013) estimate the direct healthcare costs (primary care, inpatient hospital care and day care) associated with adult overweight and obesity in Ireland in 2007 using data from 9728 adults in the nationally representative Survey on Lifestyle And Nutrition (SLAN 2007) and Quarterly National Household Survey (QNHS) Q3 2007 (*n* = 21,253). *Dee* et al. (2015) combine top-down and bottom-up cost analysis to estimate the full direct and indirect costs (healthcare, medication, productivity losses and mortality) related to adult overweight and obesity in Northern Ireland and Ireland in 2007, integrating the costs of co-morbidities attributable to weight status. Underlying data include the findings from *Doherty* et al. (2013), cross-sectional, nationally representative data from TILDA, national public hospital and reimbursement data and publicly available mortality statistics. Moreover, *Perry* et al. (2017) estimate the 2015-direct healthcare costs and projected indirect societal lifetime costs of childhood overweight and obesity in Northern Ireland and Ireland. The authors combine bottom-up cost analysis of the GUI ‘98 cohort (*n* = 7525) and top-down analysis (applying international literature to various sources of national healthcare use and prevalence data) with a closed cohort simulation model for cross-validation.

*Raftery* et al. (2012) study the individual and national direct and indirect cost of chronic non-cancer pain, and cost drivers, by collecting detailed information on healthcare use from 140 chronic pain patients randomly sampled from a national patient sample (*n* = 425) in the Prevalence, Impact and Cost of Chronic Non-Cancer Pain in Ireland (PRIME) study. Furthermore, *Gannon* et al. (2013) assess direct, indirect and informal costs related to severe chronic non-cancer pain in 100 patients attending a tertiary pain management clinic in the west of Ireland (Galway), representative of the larger chronic pain population in the PRIME study.

In the EPICure study, *Petrou* et al. (2009) assess 2006–2007-economic costs (hospital inpatient, day care and outpatient care; community health and social care, medication and (special) education services) and health utilities in eleven-year-old children born extremely preterm (*n* = 190) compared to matched term-born children (*n* = 141) in Ireland and the UK, using parental questionnaires. In a succeeding study, *Petrou* et al. (2013) assess the economic outcome (cost and utilities described above) associated with neurodevelopmental impairment or disability at 11 years of age, comparing children born extremely preterm or at term in EPICure.

*Richardson* et al. (2012) collect data on medication use from adults aged 50 years and older in the nationally representative TILDA study (*n* = 8093) to estimate the prevalence and cost of polypharmacy in the ageing Irish population and infer potential cost savings if patients switched to generic medicines.

Lastly, to estimate costs related to osteoarthritis and rheumatoid arthritis, *Doherty and O’Neill* (2014) compare self-reported healthcare use (primary care, inpatient and outpatient hospital care and A&E) in adults aged 50 years and older with these arthritic conditions to healthcare use in the same age group without these conditions, collected in the nationally representative TILDA study (*n* = 8093).

### Costing approaches and data collection

In terms of perspective, seven studies employed a societal (full [15, 17, 18, 21] and partial [22, 23, 27]), four a healthcare [19, 20, 24, 28], two a public sector [25, 26] and one a patient perspective [16]. Twelve studies employed a ‘bottom-up’ costing approach [15, 16, 18–23, 25–28] and two combined top-down and bottom-up [17, 24] (Table 2). Only four of the surveys presented in the inventory (Table 1) were employed amongst the identified literature.

For healthcare cost estimation, ten studies utilised data from a single survey [16, 19–23, 25–28]. Seven of these were dedicated ad-hoc surveys [16, 21–23, 25–27], four of which employed validated questionnaires (Client Service Receipt Inventory (CSRI) [21, 22, 27] and Resource Utilisation in Dementia Lite Instrument [23]). Of the other single-survey studies, two used TILDA [20, 28] and one employed data from the SLAN 2007 [19]. The remaining four studies employed data from a selection of surveys [18] and/or employed survey data alongside administrative data to determine utilisation [15, 17, 24] and/or supplemented survey data with estimates from small-scale, local studies in the published literature [17, 24]. All survey data integrated in cost analysis were cross-sectional and collected retrospectively [15–18, 20–28], although longitudinal data may have been partially available in a few studies (e.g. TILDA, EPICure, GUI).

Survey data were most frequently used to assess costs of primary care, including general practitioner (GP) services, (*n* = 11) [15–24, 27], followed by costs of various hospital/residential care (including outpatient and emergency hospital services, *n* = 9) [15, 18, 19, 21–23, 25–27] and medications (*n* = 8) [16, 17, 21, 22, 25–28]. Seven studies included specialist community services and other healthcare professionals [15, 17, 22, 23, 25–27] and only two studies assessed costs in each of these areas [22, 27]. While there is variability in the range of healthcare services included when relying one data source (e.g. ranging from three to six) a greater range of services can be included when a specific validated instrument like CRSI is employed, albeit across a smaller sample size and study-specific population [21–23, 27]. For example, when estimating the costs of osteoarthritis and rheumatoid arthritis, the choice of healthcare services considered was limited to those in the TILDA dataset [20].

For the various healthcare services assessed, recall periods differed across studies and sometimes within one study, ranging from one week to ten years or time since relapse or diagnosis; however most studies assessed a fixed period of six [23] or twelve months [18–20, 22, 24–27]. Where variable recall periods were assessed in one study, use of home help was assessed in the past week, medication use in the past month, diagnostic tests, outpatient visits and primary care in the past 6 months and inpatient hospital care in the past twelve months [15, 21]. One study combined six- and twelve-month recall periods due to the structure of the underlying primary data [17]. Furthermore, *Carney* et al. (2018) and *Fogarty* et al. (2014) assessed costs for home appliances and mobility and other aids over the full duration of multiple sclerosis, and *Carney* et al. (2018) assessed various costs that occurred during the last multiple sclerosis relapse. Similarly, *Ceilleachair* et al. (2017) investigated out-of-pocket costs for the full duration of colorectal cancer disease and follow-up only. *Richardson* et al. (2012) assessed medication taken daily or weekly at the time of the interview and therefore did not specify a recall period.

### Unit prices

Most studies (*n* = 11) used multiple sources to determine unit prices [15, 17–19, 21–27]. Most commonly, unit prices were informed by official national tariffs (national casemix/diagnostic-related groups) from Ireland [15, 17, 18, 21–24, 27], the UK [25, 26], or national literature [15, 17–21, 23, 24]. Studies also frequently relied on data from the Irish Central Statistics Office [15, 17, 18, 21–24], however this was mainly to determine unit prices related to productivity losses and pre-mature mortality [24]. Health professionals’ expert opinions or other cost estimates directly retrieved from primary care services, hospitals and laboratory and finance departments were also used, as well as use of national salary scales or pharmaceutical price lists and databases [15, 19, 21, 22, 24, 27]. Only few studies used information retrieved from social welfare services (e.g. national home scheme support) [18, 21], other company and service price lists [15, 21], patients’ cost estimates [16, 21], or a specialised personal social service research unit (for assessment of community health and social service unit costs) [25, 26].

### Data quality and reliability

When addressing study limitations, 13 studies referred to data issues [15–23, 25–28]. Concerns included absence of resource use data [17, 18, 28], low response rates and potential for sampling bias and incomplete data, particularly in small dedicated ad-hoc surveys [16, 21, 27], lack of suitable longitudinal/cross-sectional data to inform reliable bottom-up estimates [18, 22], reliance on dichotomous utilisation measures [19] and limited range of services [20] captured in population surveys. Issues surrounding the availability of data led researchers to employ a mix of top-down and bottom-up costing methodologies [17]. Where bottom-up only was employed, authors express concern regarding the generalisability of these results [22]. Additional concerns were highlighted relating to recall bias, which is inevitable when relying on retrospective patient surveys, rather than registry data [15, 16, 21, 27]. This was raised particularly where surveys were completed on behalf of patients [23, 25, 26]. Risk of bias due to patient self-selection was mentioned in one study that collected information from a disease-specific patient sample [27]. As a side issue, several studies highlight the lack of reference costs for Ireland [17, 22, 23] and the fragmented nature of systems preventing linking [22].

Researchers addressed some of these limitations through sensitivity and sub-analyses and by providing estimated cost ranges, in addition to single figures presented in their main analysis [17–19, 21–24, 27]. These cost ranges were typically compared to international and, where available, national literature, and reasons for potential differences between study findings and the literature were frequently explored [15–19, 21–28]. Patients with disabilities or comorbidities were explicitly excluded from some studies, or the analysis was adjusted for the added costs due to these conditions, so as not to overestimate disease-specific costs [17–19, 23]. Generally, researchers sought to use samples that were nationally or regionally representative of the wider population [15, 18, 19, 24], or of the patient group studied [15, 16, 22]. Where representation was not guaranteed, data were often weighted using national statistics or prevalence data before costs were extrapolated to the population level [15, 17–19, 21, 22, 24, 27, 28]. Additionally, bootstrapping was applied to address uncertainties related to small or unbalanced samples [17, 21, 23], and logarithmic transformations were applied to account for skewness of data [21, 22]. Where ad-hoc surveys were used, their design was often based on tested survey tools and included validated instruments (e.g. to assess disease status) [15, 17, 21, 23, 25–27]; some ad-hoc surveys were pre-piloted [22, 25–27] and mechanisms were used to increase participation (e.g. reminder letters) [15, 16, 25, 26] and completion (e.g. telephone interviews) [27]. Lastly, the interviewers of the TILDA data used in *Richardson* et al. (2012) asked participants to report and subsequently show interviewers the medication they were taking, to allow interviewers retrieve the details for all medication provided, for more accurate estimation of polypharmacy costs.

## Discussion

This scoping review identified 14 studies from 2009 to 2019 using self-reported data on healthcare use or cost to assess direct healthcare costs in Ireland. Our search identified 27 surveys that provide self-reported data on health or healthcare use; only four pre-existing population surveys and six ad-hoc surveys were used to estimate costs according to our search criteria.

Despite the inclusion of self-reported healthcare use in many Irish datasets, this review demonstrates their limited application for the estimation of healthcare costs. One explanation could be related to the concern among some researchers that existing datasets are not fit for purpose, for example due to the limited choice of healthcare services for which data are collected, therefore necessitating the supplementation with published data and dedicated data collection. However, as this review reveals, such ad-hoc data collection efforts yield small samples, which are subject to selection bias. In fact, a number of identified nationally representative datasets with large sample sizes provide data on healthcare service utilisation, along with health status, demographic, socio-economic and lifestyle information. Many of these datasets consist of repeated cross-sectional or longitudinal survey waves, but have not been subject to cost assessments in Ireland to date. This could be linked to low awareness of these datasets among researchers and health economists (representation of the latter is relatively low in Ireland); difficulties in accessing datasets through publicly available sources and limited funding opportunities for analysing existing datasets. Research funding opportunities in Ireland tend to be biased towards collecting new data. Encouragingly, however, surveys designed and administered through research institutions (e.g. GUI, TILDA) are more likely to be utilised for cost analysis; suggesting that involving (health economic) researchers early in survey design could potentially increase the usability of population health surveys for cost analysis.

The studies included in this review assess the costs of multiple sclerosis, overweight and obesity, chronic non-cancer pain, arthritis, dementia, very preterm birth, colorectal cancer and polypharmacy. This shows a tendency to non-acute, chronic conditions with a high prevalence that concern predominantly the older ages, or very young people and their future costs. It is questionable whether this implies sufficient resource coverage/provision in acute care in Ireland, or may owe to the availability (and thus awareness) of large survey data particularly for the older ages (TILDA) and children (GUI). While all authors mention gaps in data availability, there is a considerably strong emphasis on the dearth in unit cost data particularly relating to older age [17] and demand for more accurate and reliable population-based information on health service utilisation, school/work productivity and psychosocial wellbeing of children [24].

Our survey inventory revealed mixed levels of data coverage. A number of population surveys convey a multitude of questions on use of various healthcare services, demographics, health-related risk and behaviour (GUI, SPHERE, the Irish Health Survey supplement to the QNHS and Healthy Ireland); making these surveys potentially more suitable for cost assessments relative to other surveys identified. Repeated surveys that are potentially still on-going and that collected data only for some healthcare services, e.g. primary care, should consider future extension to assess resource use more comprehensively, including use of outpatient hospital services (EU-SILC, Healthy Ireland, Household Budget Survey, Irish Health Survey-QNHS, SLAN and TILDA). This is in line with data limitations highlighted in studies that used some of these surveys for cost analysis. Particularly, these studies were able to investigate GP service use [18–20, 24], inpatient hospital care [18–20], outpatient hospital services [18–20], emergency department visits [20] and medication use [28]. In contrast studies using ad-hoc surveys additionally investigated various specialist services including imaging and laboratory investigations [15, 17, 21–23, 25–27] (rehabilitation, respite care and long-term care services [15, 21, 23], mental health services [22, 23, 27], alternative therapies [22, 27] and various equipment, home modification and transport costs (including ambulance care) [15, 16, 21, 22]. Broad population surveys may accordingly benefit from the addition of an open-ended question where participants can detail the frequency and type of “other” healthcare services used during the study period.

Other (repeated) population surveys also would be more instrumental for comprehensive cost analysis if questionnaires were extended in future data collection. Namely, despite assessing healthcare use, the Household Budget Survey in its current form does not contain data on health status and lifestyle that would be needed for accurate cost analysis. Moreover, the Census and European Social Survey that are carried out routinely may be a good basis for collecting data on healthcare use and lifestyle, which is currently not the case. Similar to the estimation of healthcare use, most studies relied on a variety of sources to identify unit prices, suggesting that a central repository beyond the existing national case-mix price list is needed. Some advances continue to be made in relation to this; however more healthcare services and consideration of methodologies still need to be incorporated [29].

While providing more detail on healthcare use, ad-hoc collection of data often led to relatively small, regional samples in comparison to studies using large datasets, thus impeding the studies’ generalisability. Ad-hoc surveys sampled through national patient registers [15, 16] or from multiple healthcare service providers [27, 30] seemed to provide larger sample sizes than ad-hoc surveys sampled from individual healthcare service providers [21–23]; however effective sampling strategies are needed to reach representation and complete survey response [16]. Additionally, bootstrapping can be used to address concerns around distribution, e.g. to handle non-parametric data and small sample sizes [23]. Nevertheless, larger population surveys or centralised health system data appear preferential when representation cannot be otherwise reached.

Most studies collected information on resource use over a period of six to twelve months, with shorter periods or longer periods used for frequent use (e.g. medications and home care) or infrequent use (e.g. aids and home modifications), respectively. Studies identified these time periods as most appropriate for cost analysis and to involve a relatively low risk of recall bias based on previous research. However, all studies collected data retrospectively and risk of recall bias therefore cannot be fully ruled out. In response to this, use of diaries for prospective data collection was suggested for future studies [22], indicating the need for a more proactive approach to integrating healthcare use data in budgetary planning.

The challenges associated with lack of standardisation and interoperability of Irish health data is recognised and there are plans for reform [31, 32]. While details about the full implementation of an EHR system in Ireland are unknown [1], a data repository (eHealth Ireland Open Data Portal) providing information on health service use in Ireland has been established. However, this is limited to public health services only and currently contains a limited set of healthcare use data [33, 34]. Overall, more digital solutions to manage patient data and connect health information within and across service providers and systems are being implemented. Examples include the National Medical Laboratory Information System [35], Digital Ambulance Project [36], Primary Care IT [37] and Epilepsy Lighthouse Project [38]. Most recently, the rollout of the national COVID-19 Vaccine Information System has highlighted the need for using unique patient identifiers in healthcare services, to ensure equitable, complete and safe access to vaccinations and to enable the system’s interoperability within Europe through use of standardised vaccination certificates. Out of this need, the system draws on the existing Individual Health Identifiers that were developed on the basis of the Health Identifiers Act 2014, with the explicit aim to monitor and share personal details on health status, healthcare use and demographic information among healthcare providers and national institutions [39]. While this development shows promise to fill data gaps in the future, the implementation and integration of many individual initiatives and the national EHR system remain incomplete; thus the availability of integrated, comprehensive data for research purposes is likely to take time.

Accordingly, in Ireland and other jurisdictions with similar data restrictions, researchers continue to rely on self-reported data to conduct cost analysis. While researchers’ concerns around recall bias and incomplete cost data indicate that self-reported data are sub-optimal for cost analysis, a recent study [40] demonstrated there is little difference in precision of cost estimates derived from registry data compared to well-designed health survey questionnaires. Another study highlighted that cost accuracy is primarily driven by the type of healthcare service studied [8]. Nevertheless, a review that compares cost studies using top-down, bottom-up or mixed costing approaches would be interesting in future research.

Meanwhile, acknowledging existing gaps that should be addressed in future data collection, the identified surveys and studies in this review can be used as good-practice guides informing future survey development that enable comprehensive assessments of healthcare costs. Particularly, longitudinal and repeated cross-sectional surveys, in their combined form, can provide representative estimates of healthcare use enabling cost comparisons across age groups and time. Future surveys need to be meaningful and fit for purpose; stakeholder approaches should be adopted where possible to enhance the value of the resulting data. Finally, to avoid future underutilisation of expensive population health surveys, generated data should be in line with the Findable, Accessible, Interoperable and Reusable (FAIR) Data Principles [41].

### Limitations

Despite the use of a systematic search strategy, broad search criteria and the inclusion of both peer-reviewed and grey literature, it is possible that not all studies assessing healthcare costs using self-reported healthcare service use data were identified. While this review did not aim to retrieve a complete set of existing studies, cross-referencing and consistency checks using alternative search terms were employed to minimise the risk of bias. Similarly, despite using a broad search strategy, there is a risk that health surveys may be missing in the inventory. Therefore, we encourage other researchers to amend and publish an extended inventory if applicable. Furthermore, it should be noted that the analysis of underlying survey data in terms of their methodological quality is outside the scope of this research.

## Conclusion

This scoping review identified 27 health surveys and 14 costing studies that used healthcare use or cost data from self-reported data, to assess direct healthcare costs in Ireland. The identified surveys that have the potential for analysing healthcare costs in Ireland are collated in an inventory. Researchers in and outside of Ireland can use this inventory to review data availability in the future. In the absence of integrated EHRs and unique patient identifiers in jurisdictions like Ireland, carefully designed healthcare surveys are useful tools for cost assessments; however barriers related to awareness, access and usability of these data must be considered.

## Supplementary Information

**Additional file 1: Table S1.** Literature search terms and result. **Figure S1.** Flow chart of search process and results.

## Data Availability

All data generated or analysed during this study are included in this published article.

## Abbreviations

CSRI: Client Service Receipt Inventory
EHR: Electronic Health Records
EU-SILC: European Union Statistics on Income and Living Conditions
FAIR: Findable, Accessible, Interoperable and Reusable
GUI: Growing Up in Ireland
SLAN: Survey of Lifestyle, Attitudes and Nutrition
TILDA: Irish Longitudinal Study on Ageing
ECAD: Enhancing Care in Alzheimer’s Disease study
QNHS: Quarterly National Household Survey
PRIME: Prevalence, Impact and Cost of Chronic Pain
SPHERE: Secondary Prevention of Heart Disease in General Practice
GP: General Practitioner
PRISMA-ScR: Preferred Reporting Items for Systematic reviews and Meta-Analyses extension for Scoping Reviews
