# Supplementary material for: Assessing direct healthcare costs when restricted to self-reported data: a scoping review
Source: Health Econ Rev. 2021 Sep 16;11:35. doi: 10.1186/s13561-021-00330-2 (PMC8444520; doi:10.1186/s13561-021-00330-2)
Supplement: Supplementary file 1 — Additional file 1: Table S1. Literature search terms and result. Figure S1. Flow chart of search process and results. [file 13561_2021_330_MOESM1_ESM.docx]

**Supplementary Material**

**1. Supplementary Table 1: Literature search terms and result**

|  | **Search Terms – Pub Med** | | **N1** | **N2** | **N3** |
| --- | --- | --- | --- | --- | --- |
|  | ((cost[MeSH Terms] AND questionnaire[MeSH Terms]) AND "last 10 years"[PDat] AND Humans[Mesh]) AND (Ireland[Text Word] OR Irish[Text Word]) | | 145 | 53 | 9 |
|  | ((cost[MeSH Terms] AND “Growing Up in Ireland”) AND "last 10 years"[PDat] AND Humans[Mesh]) AND (Ireland[Text Word] OR Irish[Text Word]) | | 0 | 0 | 0 |
|  | ((cost[MeSH Terms] AND “GUI”) AND "last 10 years"[PDat] AND Humans[Mesh]) AND (Ireland[Text Word] OR Irish[Text Word]) | | 0 | 0 | 0 |
|  | ((cost[MeSH Terms] AND “Irish Longitudinal Study on Ageing”) AND "last 10 years"[PDat] AND Humans[Mesh]) AND (Ireland[Text Word] OR Irish[Text Word]) | | 1 | 1 | 0 |
|  | ((cost[MeSH Terms] AND “TILDA”) AND "last 10 years"[PDat] AND Humans[Mesh]) AND (Ireland[Text Word] OR Irish[Text Word]) | | 3  (2*) | 1  (0*) | 0 |
|  | ((cost[MeSH Terms] AND “patient experience survey”) AND "last 10 years"[PDat] AND Humans[Mesh]) AND (Ireland[Text Word] OR Irish[Text Word]) | | 0 | 0 | 0 |
|  | ((cost[MeSH Terms] AND “Enhancing care in Alzheimer’s disease”) AND "last 10 years"[PDat] AND Humans[Mesh]) AND (Ireland[Text Word] OR Irish[Text Word]) | | 2  (1*) | 1 | 1 |
|  | ((cost[MeSH Terms] AND “Children’s sports participation and physical activity study”) AND "last 10 years"[PDat] AND Humans[Mesh]) AND (Ireland[Text Word] OR Irish[Text Word]) | | 0 | 0 | 0 |
|  | ((cost[MeSH Terms] AND “CoHeart”) AND "last 10 years"[PDat] AND Humans[Mesh]) AND (Ireland[Text Word] OR Irish[Text Word]) | | 0 | 0 | 0 |
|  | ((cost[MeSH Terms] AND “Eurostudent”) AND "last 10 years"[PDat] AND Humans[Mesh]) AND (Ireland[Text Word] OR Irish[Text Word]) | | 0 | 0 | 0 |
|  | ((cost[MeSH Terms] AND “Healthy Ireland”) AND "last 10 years"[PDat] AND Humans[Mesh]) AND (Ireland[Text Word] OR Irish[Text Word]) | | 0 | 0 | 0 |
|  | ((cost[MeSH Terms] AND “Insight 07”) AND "last 10 years"[PDat] AND Humans[Mesh]) AND (Ireland[Text Word] OR Irish[Text Word]) | | 0 | 0 | 0 |
|  | ((cost[MeSH Terms] AND “Insight07”) AND "last 10 years"[PDat] AND Humans[Mesh]) AND (Ireland[Text Word] OR Irish[Text Word]) | | 0 | 0 | 0 |
|  | ((cost[MeSH Terms] AND “study of sexual health and relationships”) AND "last 10 years"[PDat] AND Humans[Mesh]) AND (Ireland[Text Word] OR Irish[Text Word]) | | 0 | 0 | 0 |
|  | ((cost[MeSH Terms] AND “contraception and crisis pregnancy”) AND "last 10 years"[PDat] AND Humans[Mesh]) AND (Ireland[Text Word] OR Irish[Text Word]) | | 0 | 0 | 0 |
|  | ((cost[MeSH Terms] AND “national time use survey”) AND "last 10 years"[PDat] AND Humans[Mesh]) AND (Ireland[Text Word] OR Irish[Text Word]) | | 17  (0*) | 7  (0*) | 1  (0*) |
|  | ((cost[MeSH Terms] AND “all-Ireland traveller health”) AND "last 10 years"[PDat] AND Humans[Mesh]) AND (Ireland[Text Word] OR Irish[Text Word]) | | 0 | 0 | 0 |
|  | ((cost[MeSH Terms] AND “psychological wellbeing and distress”) AND "last 10 years"[PDat] AND Humans[Mesh]) AND (Ireland[Text Word] OR Irish[Text Word]) | | 0 | 0 | 0 |
|  | ((cost[MeSH Terms] AND “quarterly national household survey”) AND "last 10 years"[PDat] AND Humans[Mesh]) AND (Ireland[Text Word] OR Irish[Text Word]) | | 0 | 0 | 0 |
|  | ((cost[MeSH Terms] AND “QNHS”) AND "last 10 years"[PDat] AND Humans[Mesh]) AND (Ireland[Text Word] OR Irish[Text Word]) | | 0 | 0 | 0 |
|  | ((cost[MeSH Terms] AND “Living in Ireland”) AND "last 10 years"[PDat] AND Humans[Mesh]) AND (Ireland[Text Word] OR Irish[Text Word]) | | 0 | 0 | 0 |
|  | ((cost[MeSH Terms] AND “LLI”) AND "last 10 years"[PDat] AND Humans[Mesh]) AND (Ireland[Text Word] OR Irish[Text Word]) | | 0 | 0 | 0 |
|  | ((cost[MeSH Terms] AND “income and living conditions”) AND "last 10 years"[PDat] AND Humans[Mesh]) AND (Ireland[Text Word] OR Irish[Text Word]) | | 0 | 0 | 0 |
|  | ((cost[MeSH Terms] AND “EU-SILC”) AND "last 10 years"[PDat] AND Humans[Mesh]) AND (Ireland[Text Word] OR Irish[Text Word]) | | 1  (0*) | 1  (0*) | 0 |
|  | ((cost[MeSH Terms] AND “Survey on lifestyle and attitude to nutrition”) AND "last 10 years"[PDat] AND Humans[Mesh]) AND (Ireland[Text Word] OR Irish[Text Word]) | | 0 | 0 | 0 |
|  | ((cost[MeSH Terms] AND “SLAN”) AND "last 10 years"[PDat] AND Humans[Mesh]) AND (Ireland[Text Word] OR Irish[Text Word]) | | 0 | 0 | 0 |
|  | ((cost[MeSH Terms] AND “school leavers survey”) AND "last 10 years"[PDat] AND Humans[Mesh]) AND (Ireland[Text Word] OR Irish[Text Word]) | | 0 | 0 | 0 |
|  | ((cost[MeSH Terms] AND “secondary prevention of heart disease in general practice”) AND "last 10 years"[PDat] AND Humans[Mesh]) AND (Ireland[Text Word] OR Irish[Text Word]) | | 0 | 0 | 0 |
|  | ((cost[MeSH Terms] AND “PRIME”) AND "last 10 years"[PDat] AND Humans[Mesh]) AND (Ireland[Text Word] OR Irish[Text Word]) | | 2  (0*) | 2  (0*) | 2  (0*) |
|  | ((cost[MeSH Terms] AND “chronic non-cancer pain in Irish primary schoolchildren”) AND "last 10 years"[PDat] AND Humans[Mesh]) AND (Ireland[Text Word] OR Irish[Text Word]) | | 1  (0*) | 1  (0*) | 1  (0*) |
|  | ((cost[MeSH Terms] AND “pain”) AND "last 10 years"[PDat] AND Humans[Mesh]) AND (Ireland[Text Word] OR Irish[Text Word]) | | 28  (16*) | 14  (8*) | 4  (1*) |
|  | ((cost[MeSH Terms] AND “EU-SILC”) AND "last 10 years"[PDat] AND Humans[Mesh]) AND (Ireland[Text Word] OR Irish[Text Word]) | | 2  (1*) | 0 | 0 |
|  | ((cost[MeSH Terms] AND “census”) AND "last 10 years"[PDat] AND Humans[Mesh]) AND (Ireland[Text Word] OR Irish[Text Word]) | | 1  (0*) | 1  (0*) | 0 |
|  | ((cost[MeSH Terms] AND “EQ-5D-5L”) AND "last 10 years"[PDat] AND Humans[Mesh]) AND (Ireland[Text Word] OR Irish[Text Word]) | | 1 | 0 | 0 |
|  | ((cost[MeSH Terms] AND “European social survey”) AND "last 10 years"[PDat] AND Humans[Mesh]) AND (Ireland[Text Word] OR Irish[Text Word]) | | 0 | 0 | 0 |
|  | ((cost[MeSH Terms] AND “European social survey”) AND "last 10 years"[PDat] AND Humans[Mesh]) AND (Ireland[Text Word] OR Irish[Text Word]) | | 0 | 0 | 0 |
|  | ((cost[MeSH Terms] AND “ESS”) AND "last 10 years"[PDat] AND Humans[Mesh]) AND (Ireland[Text Word] OR Irish[Text Word]) | | 0 | 0 | 0 |
|  | ((cost[MeSH Terms] AND “ESS”) AND "last 10 years"[PDat] AND Humans[Mesh]) AND (Ireland[Text Word] OR Irish[Text Word]) | | 0 | 0 | 0 |
|  | ((cost[MeSH Terms] AND “National Adult Nutrition Survey”) AND "last 10 years"[PDat] AND Humans[Mesh]) AND (Ireland[Text Word] OR Irish[Text Word]) | | 0 | 0 | 0 |
|  | ((cost[MeSH Terms] AND “National Adult Nutrition Survey”) AND "last 10 years"[PDat] AND Humans[Mesh]) AND (Ireland[Text Word] OR Irish[Text Word]) | | 0 | 0 | 0 |
|  | ((cost[MeSH Terms] AND “HBS”) AND "last 10 years"[PDat] AND Humans[Mesh]) AND (Ireland[Text Word] OR Irish[Text Word]) | | 0 | 0 | 0 |
|  | ((cost[MeSH Terms] AND “HBS”) AND "last 10 years"[PDat] AND Humans[Mesh]) AND (Ireland[Text Word] OR Irish[Text Word]) | | 0 | 0 | 0 |
|  | ((cost[MeSH Terms] AND “Household Budget Survey”) AND "last 10 years"[PDat] AND Humans[Mesh]) AND (Ireland[Text Word] OR Irish[Text Word]) | | 0 | 0 | 0 |
|  | ((cost[MeSH Terms] AND “Household Budget Survey”) AND "last 10 years"[PDat] AND Humans[Mesh]) AND (Ireland[Text Word] OR Irish[Text Word]) | | 0 | 0 | 0 |
|  | ((cost[MeSH Terms] AND “European Union Survey of Income and Living Conditions”) AND "last 10 years"[PDat] AND Humans[Mesh]) AND (Ireland[Text Word] OR Irish[Text Word]) | | 0 | 0 | 0 |
|  | ((cost[MeSH Terms] AND “EPICure”) AND "last 10 years"[PDat] AND Humans[Mesh]) AND (Ireland[Text Word] OR Irish[Text Word]) | | 1  (0*) | 1  (0*) | 1  (0*) |
| *TOTAL PubMed* | | | *205*  *(167*)* | *83*  *(63*)* | *19*  *(11*)* |
|  | | | | | |
| Grey Literature Search | | | N1 | N2 | N3 |
| Websites & Repositories  Irish Social Science Data Archive (ISSDA); safefood,  Central Statistics Office (CSO)  Department of Health (DoH) | | - Publications listed on the websites of surveys were searched for relevant titles - A text word search for ‘cost’ was performed to narrow down the list of identified papers and reports retained for analysis.   Cross-referencing/hand searching of papers and reports that met the inclusion criteria was furthermore used to identify additional studies which may not have been captured by our search strategy. | 40 | 18 | 2 |
|  | | | | | |
| Cross-referencing | | | N1 | N2 | N3 |
| Literature reviewed in full was hand-searched for additional studies | | | 2  (1*) | 2  (1*) | 2  (1*) |
|  | | | | | |
| *TOTAL ALL SEARCHES* | | | *247*  *(208*)* | *103*  *(82*)* | *23*  *(14*)* |
|  | | | | | |
| *Notes:*  N1: Number of all search results  N2: Number of studies assessing direct healthcare costs in Ireland  N3: Number of studies that meet all inclusion criteria  * number of studies after exclusion due to duplicates  MeSH Term ‘cost’ is equivalent to ‘costs and cost analysis’.  Search term ‘questionnaire’ is included, the results for which were are identical to those searching the term ‘survey’, which was confirmed through consistency checks swapping out search terms.  Surveys used in searches 2-45 were identified via Irish Social Science Data Archive (ISSDA), safefood, Central Statisitics Office and Department of Health repositories. | | | | | |

**2. Supplementary Figure 1: Flow chart of search process and results**

**INITIAL SEARCHES (n = 247)**

PubMed **(n=205)**

Grey Literature **(n=40)**

Cross-referencing **(n=2)**

## Identification

Duplicates removed (**n =39**)

Records screened – title and abstract
PubMed **(n=167)**

Grey Literature **(n=40)**

Cross-referencing **(n=1)**

## Screening

Records excluded as not eligible: data not self-reported **(n = 68)**

Not in Ireland (**n=32**);

No assessment of direct healthcare costs (**n =94**)

## Eligibility

Records for full text review

PubMed **(n=63)**

Grey Literature **(n=18)**

Cross-referencing **(n=1)**

## Included

Papers included
Systematic Search **(n =11)**

***Plus***

Grey Literature Search **(n =2)**

***Plus***

Cross-referencing **(n=1)**

Notes:

The initial literature search on PubMed retrieved 145 abstracts. Furthermore, 60 abstracts were identified in PubMed searching for individual survey names and two through cross-referencing, 39 of which were removed due to duplication. A large number of studies were conducted in Northern Ireland (n=18), the United Kingdom (n=10) or elsewhere outside of the Republic of Ireland (n=4). Furthermore, many studies reported solely on productivity losses (n=5) or indirect healthcare costs (n=8), such as those of informal caregivers, rather than direct healthcare costs. A number of studies did not assess costs at all (n=59). Furthermore, a number of studies used healthcare-provider reported data (n=26), such as patient registry data collected in the Hospital In-Patient Enquiry system, or simulation or decision-tree designs (n=2) to assess healthcare use and costs. Five studies assessed costs using relative risks or cost data from previously published literature, three studies were literature reviews and eight studies were protocols and accordingly did not collect data on healthcare utilisation or cost. Other studies collected cost data as part of a clinical trial (n=8).

Forty reports from Ireland were identified in the grey literature, 22 of which did not report direct healthcare costs and 16 of which did not fulfil other eligibility criteria.

The search string used in PubMed and a list of search results is provided in Appendix 1.
